# Drug Promiscuity in PDB: Protein Binding Site Similarity Is Key

**DOI:** 10.1371/journal.pone.0065894

**Published:** 2013-06-21

**Authors:** V. Joachim Haupt, Simone Daminelli, Michael Schroeder

**Affiliations:** Biotechnology Center (BIOTEC), TU Dresden, Dresden, Germany; Universite de Sherbrooke, Canada

## Abstract

Drug repositioning applies established drugs to new disease indications with increasing success. A pre-requisite for drug repurposing is drug promiscuity (polypharmacology) – a drug’s ability to bind to several targets. There is a long standing debate on the reasons for drug promiscuity. Based on large compound screens, hydrophobicity and molecular weight have been suggested as key reasons. However, the results are sometimes contradictory and leave space for further analysis. Protein structures offer a structural dimension to explain promiscuity: Can a drug bind multiple targets because the drug is flexible or because the targets are structurally similar or even share similar binding sites? We present a systematic study of drug promiscuity based on structural data of PDB target proteins with a set of 164 promiscuous drugs. We show that there is no correlation between the degree of promiscuity and ligand properties such as hydrophobicity or molecular weight but a weak correlation to conformational flexibility. However, we do find a correlation between promiscuity and structural similarity as well as binding site similarity of protein targets. In particular, 71% of the drugs have at least two targets with similar binding sites. In order to overcome issues in detection of remotely similar binding sites, we employed a score for binding site similarity: LigandRMSD measures the similarity of the aligned ligands and uncovers remote local similarities in proteins. It can be applied to arbitrary structural binding site alignments. Three representative examples, namely the anti-cancer drug methotrexate, the natural product quercetin and the anti-diabetic drug acarbose are discussed in detail. Our findings suggest that global structural and binding site similarity play a more important role to explain the observed drug promiscuity in the PDB than physicochemical drug properties like hydrophobicity or molecular weight. Additionally, we find ligand flexibility to have a minor influence.

## Introduction

### Drug Promiscuity

Not too long ago, a drug binding to multiple different targets seemed to be more the exception than the rule and was unwanted in drug development due to possible side effects. Thus, the pharmaceutical industry focused on the development of highly selective single-target drugs. However, the high attrition rates in late stage clinical trials due to a lack of efficacy [1] indicate that something is wrong with the “one drug – one target” paradigm. Now, it is clear that polypharmacology – also termed as drug promiscuity – is not only widespread [2], but also important for the efficacy of drugs [3]. A promiscuous drug can be both, a curse and a blessing. Undesired side effects are inter alia due to the binding of drugs to off-targets. On the other hand, this gives the opportunity to uncover new uses for already known drugs [4], [5] and increase the efficacy of drugs [6], as reported for antipsychotic or anticancer drugs. In particular, there are efforts to develop promiscuous drugs, especially for complex diseases [3]. Approaches to discover new drug targets and uses are manyfold [7], ranging from the analysis of genome wide association studies [8], gene expression data [9] and networks [10]–[12] to structural approaches [5], [13]. Structural binding site comparison approaches can be distinguished in alignment methods [14]–[16] and alignment-free methods, e. g. using finger prints [17], [18]. The latter have the advantage of uncovering also distant similarities with great success but do not provide an aligned structure.

Despite the importance of drug promiscuity, there is still an open debate regarding its underlying reason and its definition.

### Promiscuous Drugs: Hydrophobicity vs. Molecular Weight

Over the past ten years, researchers mainly focused on drug properties such as hydrophobicity and molecular weight as explanation for promiscuity. Table 1 summarizes nine studies, primarily by pharmaceutical companies, comprising up to 75000 drug like compounds and over 500 targets. However, their comparison reveals inconsistency since they draw contradictory conclusions.

**Table 1:** Overview of Drug Promiscuity Studies.

Organization	Drugs	Targets	Hydrophob.	MW
Pfizer [20]	75000	220	yes	low
UCSF (USA) [30]	70563	–	artifact	
EBI [25]	40408	>500	yes	high
Novartis [19]	3138	79	yes	high
GSK [26]	2500	490	yes	indep.
AstraZeneca [22]	2133	200	yes	indep.
IMIM/UPF (Spain) [24]	802	480	yes	low
Roche [23]	213	–	yes	indep.
Organon [21]	138	–		low

Controversy over drug promiscuity: hydrophobicity and molecular weight.

Azzaoui *et al*. [19] reported that promiscuity – computed by a model based on naïve Bayesian classification on 3138 compounds and 79 targets – correlates with molecular weight. I.e. highly promiscuous drugs have a high molecular weight and drugs with low molecular weight are weakly promiscuous. Additionally, they found that hydrophobicity (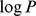) and the number of nitrogen atoms are higher while the number of oxygen atoms is lower for promiscuous drugs. Moreover, they found that marketed drugs are more selective and hence less promiscuous.

Hopkins *et al*. observed the opposite when analyzing Pfizer screening data of 75,000 compounds and 220 targets: An inverse correlation between molecular weight and promiscuity [20] (i.e. the higher the mean molecular weight of a compound, the lower the promiscuity). They also found hydrophobicity to be higher for promiscuous compounds [20]. Morphy *et al*. (Organon) observed a similar trend regarding molecular weight [21]. The study of BioPrint (AstraZeneca, 2,133 compounds and 200 targets) showed similar results regarding hydrophobicity but found no general correlation between molecular weight and promiscuity [22]. Peters *et al*. investigated 213 Roche compounds. The results agreed with the aforementioned studies concerning hydrophobicity [23]. They additionally reported that a positive charge in drug molecules increases their potential for promiscuity. Regarding a relationship between promiscuity and molecular weight, they could not find a correlation [23], resulting in a compromise between the conclusions of the aforementioned studies.

Another study analyzed seven databases of drug-target interactions, showing that promiscuity drops with increasing molecular weight and – in agreement with the other studies – that promiscuity increases with increasing hydrophobicity [24]. Recently, a study on the ChEMBL database by Gleeson *et al*. tried to sort these controversial results. By dividing the number of micromolar potencies by the total number of reported activities, they showed that high potency promiscuity increases with molecular weight [25]. Using this normalization, they also found – regarding hydrophobicity – the consensus trend of the aforementioned works. In addition, they observed that neutral or basic molecules are more promiscuous than acidic molecules [25]. The very recent analysis of Leach and Hann of 2500 GlaxoSmithKline compounds showed that promiscuity correlates with hydrophobicity, but molecular weight does not significantly change within certain hydrophobicity ranges [26].

The overall conclusion of these studies is that the more hydrophobic a drug is, the more likely it is to be promiscuous. Due to the non-selective characteristics of hydrophobic interactions, drugs with such properties may also accumulate at lipid bilayers and thus interact with signaling molecules [26].

A clear relationship between molecular weight and drug promiscuity was not apparent in the existing studies. Hann *et al*ifnextchar. found that the probability of a ligand binding to a protein drops with increasing ligand complexity because the selectivity of the ligand increases due to the higher number of chemical features [27]. This could explain the observation of higher molecular weight implying lower promiscuity. For the converse observation (higher molecular weight implies higher promiscuity), a relaxed version of this model (allowing unmatched entities) can be applied [26]. Thus, the probability of a complex ligand to interact with a binding site increases due to a higher number of possible matching interaction features, whereas other features are not matched. Clearly, this leads to a decrease of potency [26]. However, it is questionable whether an unmatched portion of a ligand (eventually having a considerable portion exposed to the solvent) is sufficient to establish more than a short-term binding event. Additionally, a binding affinity threshold of 10 µM (as applied in most of the aforementioned studies) will also reflect unselective binding due to hydrophobic interactions or aggregation.

Indeed, the screening libraries of pharmaceutical companies may be biased by promiscuous inhibitors acting by aggregation [28]–[30]. Moreover, the compounds forming aggregates were found to be typically hydrophobic [28], reflecting the increase in promiscuity with an increase in hydrophobicity.

To summarize, researchers have focused on molecular weight and hydrophobicity as explanation for promiscuity. Regarding both explanations, contradictory studies can be found. Not all studies do find a correlation with molecular weight, but all do find a link to hydrophobicity. However, Feng *et al*. [30] show that this may be an artifact resulting from drug aggregation.

### Ligand Flexibility vs. Binding Site Similarity

The studies discussed above were mostly performed by large pharmaceutical companies and are based on large compound screens. All of the above screens are drug centric and thus omitting properties of the drug targets as explanation for promiscuity. Here, we address this problem by following a structural approach based on protein-drug complexes from the PDB (Protein Data Bank [31]).

We present a systematic study of all protein structural data available to shed light onto the source of drug promiscuity, contributing to the above controversy from a structural point of view. Besides basic drug physicochemical properties like hydrophobicity and molecular weight, as in the studies above, two additional explanations – in the context of drug promiscuity in the PDB – are analyzed: The flexibility of promiscuous drugs and the binding site similarity among their targets.

These properties are potential explanations for drug promiscuity, since a drug might be promiscuous because it is flexible and thus can adapt to multiple different targets. On the other hand, a promiscuous drug might be able to bind to different receptors because their binding sites are similar in terms of their shape and physicochemical properties.

**Figure 1:**
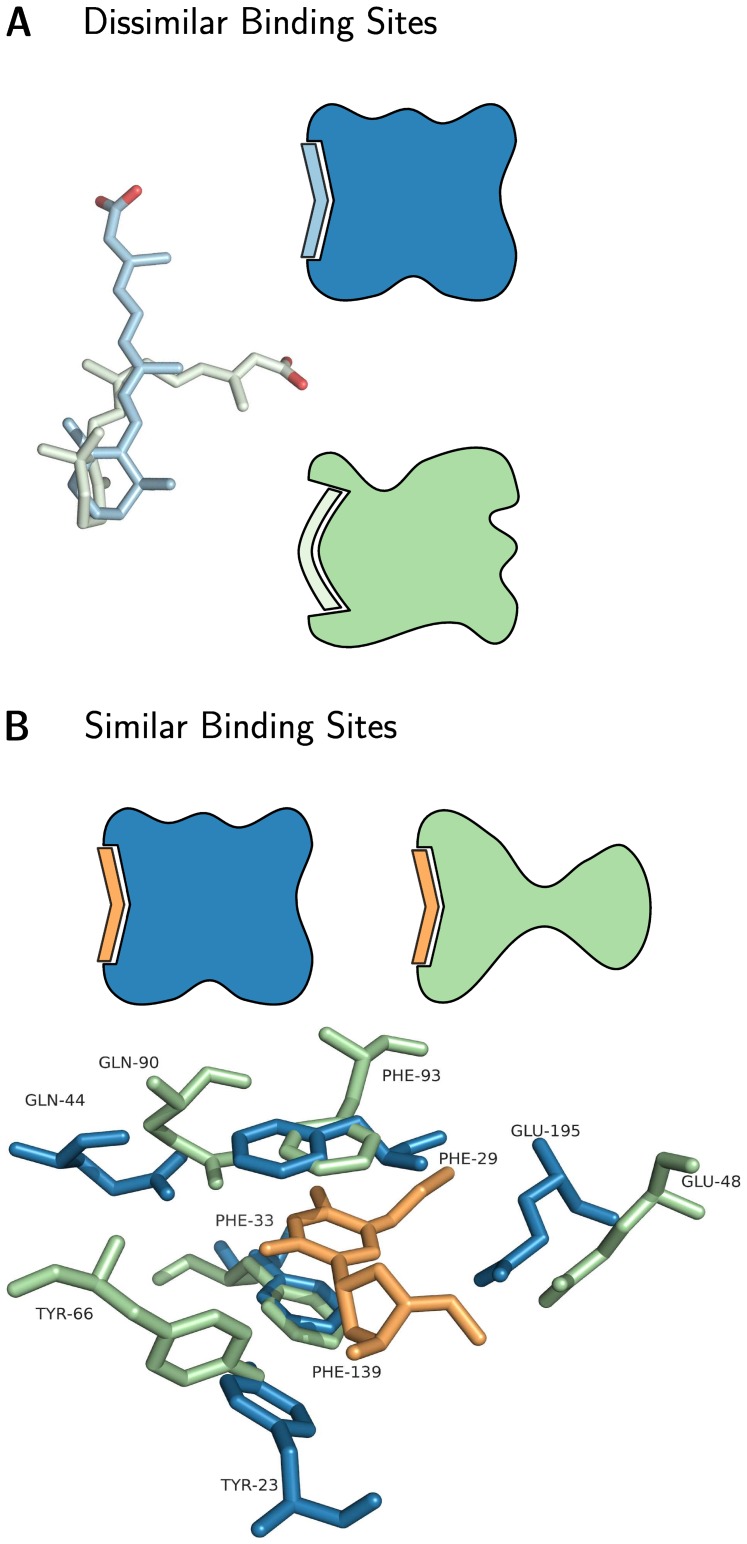
Drug promiscuity: Ligand flexibility vs. binding site similarity. (**A**) A flexible ligand, tretinoin (on the left), with two distinct conformations is able to bind to very different binding sites. (**B**) The drug BVDU (orange) binding to a viral thymidine kinase (green, 1osn) and a human heat shock protein (blue, homology model [32]). The two targets share a similar binding site, which allows the promiscuous binding of the drug in the same conformation.

To illustrate these hypotheses, consider Figure 1. The flexibility of the drug ligand tretinoin – a drug marketed for skin diseases is visualized in Figure 1.A. Tretinoin adapts to the two conformers to bind to the alpha helical retinoid X receptor and to lipocalin, a beta barrel.

Figure 1.B shows BVDU, an anti-herpes drug which has been on the market since the 1980s, being recently repositioned to tackle chemoresistance in pancreatic cancer [32]. BVDU binds to the herpes virus thymidine kinase and to the human heat shock protein Hsp27, which is involved in chemoresistance in pancreatic cancer. Despite the two targets are completely unrelated, the binding sites in thymidine kinase and a model of Hsp27 have five residues at similar positions, including two key phenylalanines that coordinate the drug by pi-stacking (Figure 1.B), as was experimentally validated [32].

Overall, Figure 1 shows that, in principle, both ligand flexibility as well as binding site similarity can serve as hypotheses to explain drug promiscuity from a structural point of view. In the remainder of the paper, we screen all relevant protein structures and evaluate four potential explanations for promiscuity – hydrophobicity, molecular weight, ligand flexibility and binding site similarity – based on drug-protein target complexes from the PDB. The available data is being correlated with the degree of drug promiscuity (i.e. number of targets) and physicochemical properties distributions for promiscuous PDB drugs are compared to all drugs in the PDB.

## Results and Discussion

### Drug Promiscuity

Drug promiscuity is receiving a lot of attention [19]–[26], [30]. Often, authors consider a drug promiscuous if it binds more than one target (e. g. [20], [33]). However, especially in large screens, drug targets are not defined precisely [25], [34]. So, under which circumstances are targets different? A fine grained view considers two targets of a drug different if they have less than 95% sequence identity. A granular view considers two targets of a drug different if they fall into different Pfam families (and thus would have less than ca. 30% sequence identity). We present both approaches and provide the corresponding results. For the granular view, we use Pfam [35] and for the fine grained view, we cluster proteins at 95% sequence identity.

The clustering of protein targets at 95% sequence identity gives a non-redundant target set for the present study. To ensure that these targets are not highly similar, we have systematically computed pairwise target sequence similarity (see Figure S1). As the figure shows, the targets cover the full range of sequence identity with a mean of 18% and a median of 12%. Besides shedding light on the targets of promiscuous drugs by evaluating the targets’ sequence similarity, their global structural similarity, their protein family membership, and their binding site similarity are investigated. The first three measures will help to assess how targets relate globally, while the last is the focus of this paper since it addresses the exact mode of binding. Among the first three, the evaluation with Pfam helps to distinguish different flavors of promiscuity such as the example of staurosporine (targeting different kinases) and BVDU discussed above.

### Drug-Target Dataset

To create a meaningful structural drug-target data set, we proceeded as follows: Starting from an integrated dataset of 3551 drugs from the Therapeutic Targets Database (TTD), the Comparative Toxicogenomics Database (CTD), and DrugBank, we selected 543 drugs being present in PDB structures and 164 of those with three or more targets in the PDB. These drugs bind a set of 712 non-redundant protein structures (clustered by 95% sequence identity, see Methods).

To assess the strength of the drug-target interactions in PDB, we compared our drug-target pairs to affinity data from BindingDB [36]. Overall, the mapped ligands (6% of the drug-target pairs are covered) bind with high affinity: 46% of these bind at least in the range, 72% bind in the µM range or better and 98% bind in the range or better (data not shown). However, promiscuous drugs do not necessarily have to bind with high affinity since the low-affinity binding of multiple targets also leads to high efficacy [37].

Our dataset is limited in terms of coverage of the chemical space of drugs as well as in terms of coverage of the protein space. Important drug targets like GPCRs and other membrane proteins [34] are underrepresented in the PDB due to their hydrophobic nature. However, the PDB is estimated to cover the vast majority of the known drug targets (92% when considering similar proteins) [34]. Moreover, the screens listed in Figure 1 are considerably larger compared to our set of 164 promiscuous drugs, since they do not consider structural data. Thus, the analysis in this study must be interpreted with these limits in mind.

**Figure 2:**
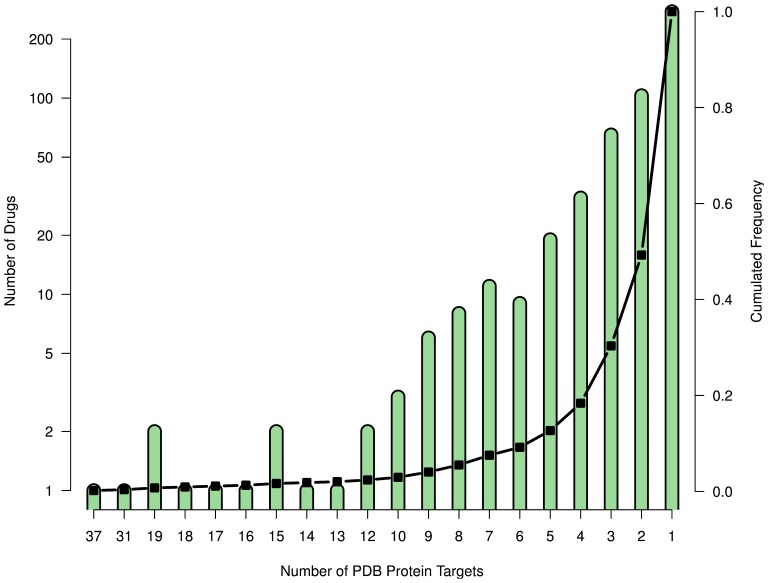
Promiscuity of drugs in the PDB. 30% of the drugs have three or more targets.

Figure 2 summarizes the distribution of drug promiscuity in PDB. The majority of drugs has only one target, but some 30% (164) have three or more targets (see Methods for details). The top 10 of these promiscuous drugs are listed in Table 2. The most promiscuous drug with 37 different targets is benzamidine, a competitive inhibitor of serine proteases and a precursor of many drugs such as pentamidine. However, it is overrepresented since it is used in X-ray crystallography to prevent degradation of proteins. Second is staurosporine, a non-selective kinase inhibitor with 31 targets having a sequence identity down to 3% (average 23%). Derivatives of staurosporine are in clinical trial for different cancers. The first three promiscuous drugs that are on the market are acarbose (diabetes), methotrexate (cancer and auto-immune diseases), and niacinamide (skin diseases). Each has over 15 distinct targets with average pairwise sequence similarities of 12%, 18%, and 10%, respectively.

**Table 2:** The 10 most promiscuous drugs.

Organization	Targets	Approved
Benzamidine	37	
Staurosporine	31	
NANA	19	
Sinefungin	19	
Acarbose	18	**√**
Methotrexate	17	**√**
Niacinamide	16	**√**
5′-methylthioadenosine	15	
Quercetin	15	**√**
Tretinoin	14	**√**

NANA stands for 2-deoxy-2,3-dehydro-N-acetylneuraminic acid.

### Molecular Weight does not Correlate with Promiscuity

As discussed in the introduction, there is a debate on the relation of molecular weight (MW) and promiscuity. MW is of interest in the context of promiscuity because it approximates molecular size. Since bigger molecules bare more features to interact with a receptor, they could be potentially more promiscuous [26]. On the other hand, big and thus complex molecules could be very selective due to specific interaction patterns with the receptor [27].

**Figure 3:**
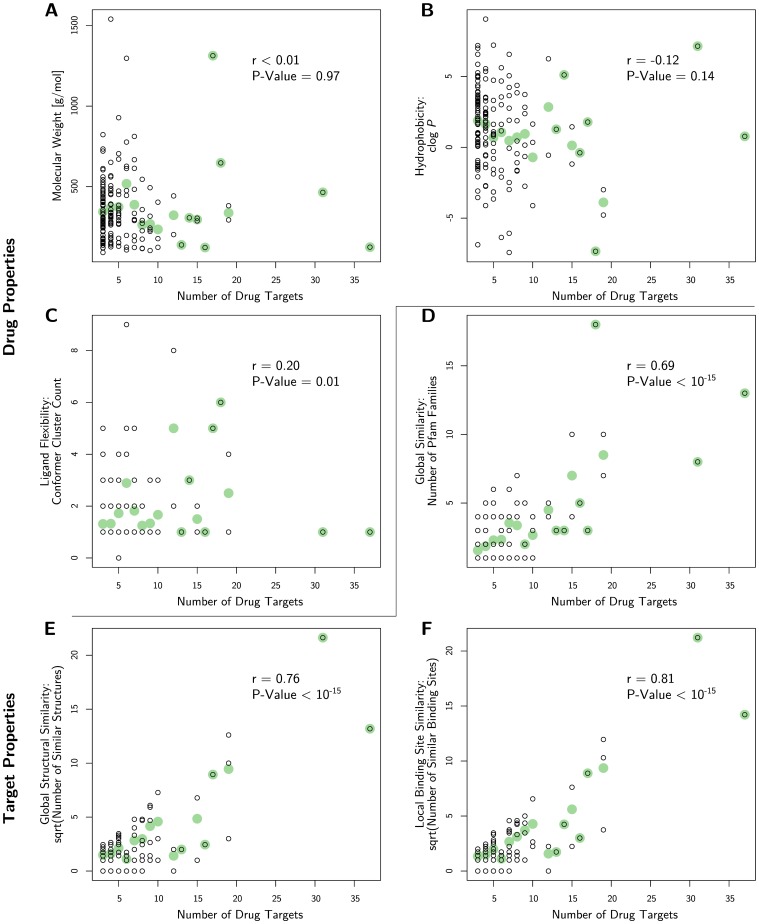
Visualization of possible correlations with degree of drug promiscuity (drug target count). Green dots denote the mean. (**A**) Molecular weight. There is no correlation (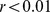). (**B**) Hydrophobicity. There is no correlation (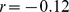). (**C**) Bound Drug conformer clusters. There is a weak correlation (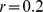). (**D**) The number of target Pfam families is correlated with the drug target count (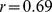). (**E**) Global structural alignment. There is a correlation between the number of targets of a drug and the square root of the number of structurally similar proteins among its targets (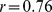). (**F**) Similar binding sites. There is a correlation between the target count of a drug and the square root of the similar binding site count of its targets (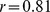).

Figure 3.A plots this relationship for the 164 promiscuous drugs of this study. Molecular weights range from 91 g/mol (aminooxyacetic acid) to 1541 g/mol (cobamamide). The top two promiscuous drugs have a MW of 124 g/mol for benzamidine and 465 g/mol for staurosporine, respectively. Overall, there is no correlation between weight and the degree of promiscuity (Pearson correlation coefficient 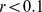). In order to test the statistical significance of the correlation, we computed P-values (see Methods). The correlation of promiscuity to molecular weight has an insignificant P-value of 0.97. Additionally, we compared the distributions of MW for the promiscuous drugs dataset and for all drugs in the PDB. These distributions were fairly similar as well (Kolmogorov-Smirnov test, distributions are similar with a P-Value of 0.58, see Figure S2). Moreover, the distributions equal the weight distribution for all PDB ligands [38], further supporting the finding that MW has no impact on the degree of promiscuity.

Thus, our data supports the findings of [22], [23], [26] that molecular weight is not indicative of promiscuity and is not in accordance with the findings presented in [19]–[21], [25].

### Hydrophobicity does not Correlate with Promiscuity

While disagreeing on the influence of molecular weight, all studies but two [28], [30] agree on hydrophobicity as a reason for promiscuity. Hydrophobicity is a fundamental criterion during drug development, since it influences e. g. solubility, membrane permeability and thus bioavailability with a direct impact on potency [39].

For the promiscuous PDB drugs, we analyzed hydrophobicity as computed octanol-water partition coefficient (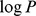). Overall, hydrophobicity varies greatly. For example, the two most promiscuous drugs, benzamidine and staurosporine have a 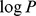 of 0.77 (slightly hydrophobic) and 7.14 (hydrophobic), respectively. Among all the promiscuous drugs, the greatest difference in hydrophobicity is between the compounds kanamycin and vitamin K1 with 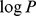 values of −7.14 and 9.06, respectively. All in all, Figure 3.B shows that there is no correlation between the number of targets and hydrophobicity (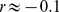, 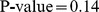).

Regarding distribution, hydrophobicity (computed 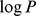) is normally distributed around 0 for all PDB ligands [38], whereas the restriction to the promiscuous PDB drugs shows a trend of higher hydrophobicity (higher 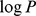, Figure S3). Hydrophobicity is a general property of drugs since protein drug targets tend to have more apolar amino acids in their binding sites than non-drug targets [40]. To assess, whether this trend is a property of the promiscuous drugs dataset, we compared the 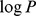 distribution for the promiscuous drugs to the distribution of 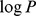-values for all PDB drugs in our dataset. The two distributions are not significantly dissimilar (P-Value of 0.13, see distributions in Figure S3). Thus, no trend of higher/lower hydrophobicity for the promiscuous drugs in comparison to all drugs in PDB is apparent.

The predominance of hydrophobic compounds among the promiscuous drugs in the studies summarized in the Introduction has been explained by works demonstrating that the observed promiscuity is due to the formation of hydrophobic aggregates [28], [30]. However, the drug dataset in this study does not sample the entire chemical space since important drug targets like GPCRs [34] and other membrane bound proteins are clearly underrepresented in the PDB.

### Determinants of Promiscuity: Drug Flexibility and Binding Site Similarity

As the analyses above suggest, there is no correlation between drug promiscuity and molecular weight or hydrophobicity. As shown in Figure 1, two reasons why a drug may be promiscuous are of structural nature: Either the drug may be flexible and can adapt to different binding sites or the binding sites of the targets are similar.

### Drug Flexibility Weakly Correlates with Promiscuity

The last drug property open for analysis is conformational flexibility. It is of interest to analyze ligand flexibility because a flexible ligand might be able to adapt to different receptors (see Figure 1) and thus being more promiscuous. Since flexibility itself cannot be measured directly, two approximations are studied. We analyze the rotatable bond count and compare all conformers (as found in the PDB structures) for each promiscuous drug.

### Rotatable Bond Count

A commonly used approximation for conformational flexibility is the rotatable bond count. Instead of using the absolute number of rotatable bonds, the relative number of rotatable bonds was used to avoid bias towards molecule size (approximated by MW). This is because the number of rotatable bonds correlates well with MW (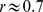, compare also Figure S4 to Figure 3.A). In contrast, the relative number of rotatable bonds is only weakly correlated with MW (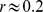).

For the promiscuous drugs, the median of the relative rotatable bond count is 0.2. Between 11% and 30% of the bonds are rotatable for half of the drugs (see Figure S5). No correlation was apparent between the relative number of rotatable bonds of the promiscuous drugs and the observed promiscuity (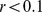, Figure S6).

### Drug Conformers

To reliably assess the influence of the ligands’ flexibility on their promiscuity, we additionally performed an analysis of the exhibited drug conformers. Thus, all conformers of the promiscuous drugs were extracted from the corresponding PDB structures and clustered to determine the different conformer counts for each drug. Conformers with an RMSD of ≤1.4 Å were clustered (see Methods and Figure S7 for the histogram of the RMSDs).

As shown in Table 3, the most flexible drug (with a maximum number of different conformers) is suramin (9 conformers). Suramin is an antiparasitic drug (against trypanosomiasis and onchocerciasis) developed in 1916 by Bayer and currently studied for its activity against various cancer cell lines [41]. It is found in 7 PDB structures (representing 6 distinct targets) in 9 different conformers. However, 3 different conformers were solely found in a single PDB structure of the toxic component of a snake venom (PDB ID 3bjw) [42].

**Table 3:** The most flexible drugs.

Drug	Targets	Conformers	Min	Mean	Max
Suramin	6	9	1	2.22	4
Farnesyl Pyrophosphate	12	8	1	4.50	22
Acarbose	18	6	1	7.33	19
Methotrexate	17	5	1	14.40	56
Glutathione Disulfide	6	5	1	1.80	3
Cholecalciferol	3	5	1	1.80	3
β-Methylene TAD	7	5	1	3.40	6
Ampicillin	5	4	1	2.50	6
5,6,7,8-TetrahydrofolicAcid	6	4	1	2.25	5
Sinefungin	19	4	1	8.25	24
Dodecyl Sulfate	3	4	1	4.25	11

Drugs with ≥4 conformer clusters. For each drug, the total number of clusters (i.e. the number of conformers in all PDB structures) and the minimum/maximum/average number of cluster members (i.e. similar conformers of one drug) in such a cluster is given.

Five different conformers were found for methotrexate, but its targets are highly similar in structure and binding site, building essentially one cluster. In terms of sequence, the methotrexate targets are clustered into three clusters.

The situation for acarbose is the converse: It shows six different conformers, which is reflected in the 5 clusters of different global structures and 9 binding site clusters. The diversity in sequence is even higher, leading to 12 sequence clusters. See the last subsection of Results/Discussion for details on methotrexate and acarbose.

**Figure 4:**
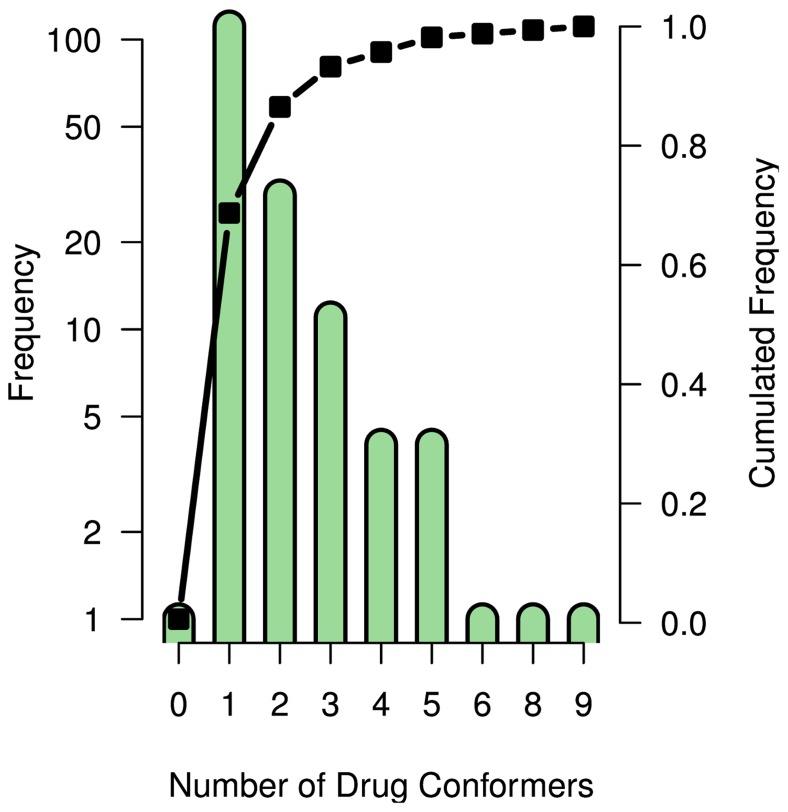
Conformer count of promiscuous drugs in the PDB.

However, these examples of highly flexible drugs are exceptions in the data set. Figure 4 summarizes the number of conformers for all drugs in a histogram. By far the most drugs (68%) show exactly one conformer when bound to a PDB protein. This is a similar result as in [43], where the authors found this number to be 54% on a set of 193 drugs. Furthermore, of the 164 drugs in our dataset, 18% showed two conformers and only 14% three or more. For one drug (pepstatin), the number of conformers was not determined because it got changed from a ligand (PDB Chemical ID: IHN) to a protein chain in the PDB structure as the analyses were performed.

Since 50% of the drugs have more than two targets (Figure 2), the conformational flexibility can not completely explain the observed promiscuity. This finding is further supported by a weak correlation between the degree of promiscuity of a drug and the number of conformer clusters (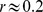, P-Value  = 0.01, Figure 3.C). Probably, the reason for the relatively low number of conformers for the promiscuous drugs lies in drug design: rigid compounds are preferred over flexible ones, reducing entropy loss upon target binding and membrane permeation [44]. The correlation between the conformer count of the drugs and their relative rotatable bond count is weak (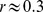), reflecting the discrepancy between the number of theoretically possible conformers of a drug and the actually observed number of conformers in the binding sites.

Comparing the distributions of the relative rotatable bond count for the promiscuous drugs and all drugs in the PDB, slight differences are apparent (P-Value of 0.007, see Figure S5). As the data suggests, promiscuous PDB drugs show a tendency towards a higher rotatable bond count in comparison to the set of all drugs in the PDB. Thus, ligand flexibility might explain the permissive binding of some drugs to highly dissimilar pockets in distinct targets.

A combination of the three studied drug properties was not related to the degree of promiscuity as resulted from an appropriate linear regression model (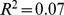 at a P-Value of 0.04).

### Binding Site Similarity Analysis

To investigate whether a pair of proteins (being targeted by a promiscuous drug) has a similar binding site, their structures are aligned locally with SMAP [15], [45], [46]. The binding site alignment essentially works by selecting the 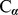 atoms from each protein and trying to find an optimal local superposition of these atoms in space, while taking their side chains’ physicochemical properties into account [5]. Since only binding sites of identical promiscuous drugs are aligned against each other, we can use the ligand positions in an aligned pair of proteins to judge the alignment. This is done by measuring distances between the atoms of the two ligands (root-mean-square deviation, RMSD). Similar measures have been applied in protein-protein docking [47], drug target identification [11] and binding site similarity assessment [46], [48]. We enriched the approach described in [48] with an automated substructure search (Small Molecule Subgraph Detector [49]), generating a robust scoring for binding site similarity: LigandRMSD (see Methods).

**Figure 5:**
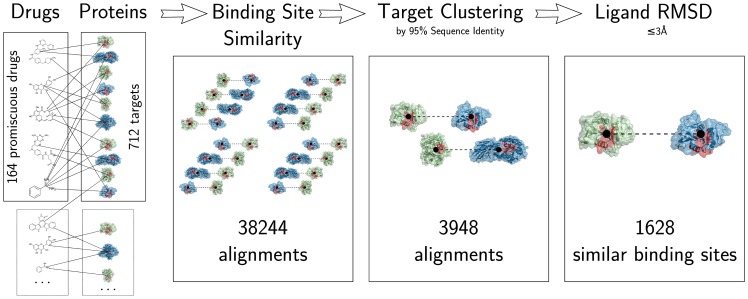
Pipeline of the binding site similarity analysis. Starting from 543 drugs, we identify 164 promiscuous drugs, each binding to three or more non-redundant targets (712 in total). The binding site alignment with SMAP is performed for all 2284 structures (i.e. the redundant targets). Subsequently, target pairs are clustered by 95% sequence identity – giving 712 non-redundant targets – and ranked with LigandRMSD.

To assess, whether promiscuous drugs have targets with similar binding sites, we implemented a pipeline consisting of three steps (see Figure 5):

1. First, we align all pairs of binding sites for all promiscuous drugs using SMAP [15], [45], [46].
2. Second, we remove redundant targets.
3. Third, we only keep sites with a consistent binding mode of the ligand.

The purpose of step one is to systematically compare any possible match of any binding sites of a promiscuous drug. Since the comparison is pairwise, the number of 712 non-redundant targets (2284 structures) leads to a total 38244 aligned binding site pairs. Some of the aligned binding sites are very similar. To remove this redundancy, all targets with ≥95% sequence identity are clustered, reducing the dataset to nearly 10% (3948 pairs). The final step is the crucial one. Since we want to study binding sites in the light of drug promiscuity, it is vital that the compared binding sites bind the ligand in a similar mode. The first step only considers the binding sites and not the ligands in its alignment. Hence, the third step, in which the ligands are compared, is necessary. This third step is called LigandRMSD since we compute how well the two ligands of the compared binding sites are aligned due to the superposition of the binding sites. This is done by measuring the RMSD of the ligand superposition and comparing it to a corresponding optimal superposition. An alignment of two binding sites was judged successful if the positions of their shared ligand in each site are similar (i.e. the protein structural alignment led to a superposition of the ligands). Therefore, two binding sites were considered similar if their alignment yields a LigandRMSD ≤3 Å (see Methods and Figure S8 for the histogram of all computed LigandRMSDs). LigandRMSD is independent of conformational differences between the ligands since it compares the alignments of the ligands against the corresponding optimal alignment. The third step of requiring consistent ligand binding reduces the set by 59%.

**Figure 6:**
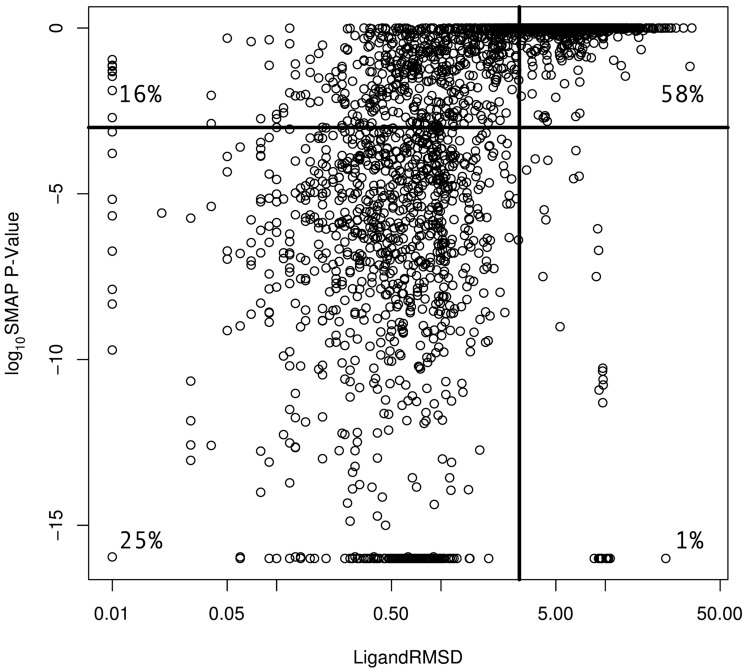
Comparison of the SMAP P-Value to LigandRMSD. A P-Value of 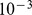 gives a significant binding site alignment. The LigandRMSD gives the conformational similarity between the bound ligands and is ≤3 Å for similar binding sites. The thresholds are displayed as solid lines in the plot. In total, 3948 non-redundant target pairs were compared.

This final set of 1628 binding site pairs satisfies now three properties: First, the binding sites share some similarity as obtained by SMAP, they are non-redundant, and they bind their ligands similarly (LigandRMSD). The current pipeline uses all binding site comparisons provided by SMAP as they are independent of their score. Thus, the question arises, whether setting up a threshold on the SMAP score could make the final step of filtering for consistent ligand conformation unnecessary. This amounts to the question whether SMAP P-Values and LigandRMSD separate the similar binding site pairs from the non-similar equivalently. To test this, we plotted the SMAP P-Value against the LigandRMSD for the corresponding pairs in Figure 6. The filtering by LigandRMSD detected more similar binding site pairs in comparison to the SMAP P-Value at any threshold without increasing the false positive count considerably. At a P-Value threshold of 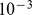, LigandRMSD detects 16% more similar binding sites. At the same time, basically all highly significant similar binding site pairs according to the SMAP P-Value are retained by the LigandRMSD filtering.

Thus, the two separations are different and hence both steps – one and three – are necessary. Intuitively, this is also supported since SMAP compares only the targets and not the ligands. While LigandRMSD focuses solely on the ligand. For this specific analysis of drug promiscuity, both perspectives are needed.

### Similar Binding Sites and Structural Similarity do Correlate with Promiscuity

1628 out of the 3948 target pairs (41%) have a similar binding site according to the alignment by SMAP together with the scoring with LigandRMSD. The average sequence identity of these 1628 target pairs is still low with 28% and the majority (1112 pairs) has less than 30% sequence identity. Taking a drug-centric view, we find that 71% of the drugs have at least one target pair with a similar binding site and that for 18% of the drugs all of their targets are similar. The top 4 promiscuous drugs (Table 4) benzamidine, staurosporine, NANA and sinefungin are also the ones with most similar binding sites among their targets (see Table 4). To check for a relation between drug promiscuity and binding site similarity, Figure 3.F shows a plot of the degree of promiscuity against the square root of the number of similar binding sites. The square root is taken since there are potentially 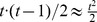 similar binding sites for 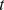 targets. Overall, Figure 3.F shows a correlation of 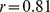 (P-Value 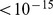).

**Table 4:** The 10 drugs with the most similar binding sites.

		Sequence Identity					
Drug	Sim. BS	min	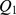	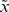	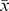	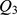	max
Staurosporine	450	3	19	22	23	25	80
Benzamidine	202	0	27	33	32	37	90
NANA	143	2	9	13	15	17	70
Sinefungin	106	4	9	12	12	15	27
Methotrexate	79	4	31	26	26	31	89
5′-methylthioadenosine	58	1	10	14	14	16	45
Actinonin	43	24	27	29	36	49	70
5,6,7,8-tetrahydrobiopterin	25	12	60	64	63	67	92
1-Methyl-3-isobutylxanthine	25	20	26	27	29	29	92
Zanamivir	21	2	12	29	31	47	92

For each entry, the minimum, lower (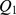) and upper quartile (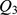), the median (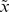), average (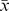) and maximum sequence identity (among pairs with similar binding sites) is given. NANA stands for 2-deoxy-2,3-de-hydro-N-acetyl-neuraminic acid.

It must be noted that any found binding site similarity is significant and *a priori* unlikely since the average sequence identity of all compared target pairs sharing a drug is just 19% (Figure S1). Furthermore, 3310 out of the 3948 compared non-redundant target pairs have a sequence identity of less than 30%. The distributions in Figure S1 show that the targets are dissimilar in sequence (sequence identity ≤23% for half of all pairs), although they are similar in binding site.

To assess the influence of global structural similarity on the detected similar binding site pairs, global protein structural alignments were computed using TM-align [50] in the same way as for SMAP. Thus, the global structural alignments were also filtered with LigandRMSD. Figure S9 shows the distribution of the TM-scores for the aligned proteins. 55% of these protein pairs are dissimilar in global structure with a TM-score<0.5, demonstrating the diversity among a drug’s targets. The correlation of global structural similarity (TM-score≥0.5) with the degree of promiscuity was with 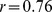 (P-Value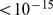, Figure 3.E) weaker than for binding site similarity with SMAP (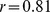, Figure 3.F). Moreover, 15% of the similar binding site pairs are significantly dissimilar in global structure with a TM-score<0.5. Such cases of binding site similarity in the absence of structural and sequence similarity are of particular interest, since they would not be discovered by conventional methods; although being potential off-targets causing severe side effects.

Furthermore, we investigated the diversity in the dataset of similar binding site pairs in terms of their family membership using Pfam. However, 56% of the promiscuous drug targets did not have a Pfam annotation. We found a strong correlation (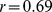, P-Value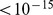, Figure 3.D) of the different family count with the degree of promiscuity, demonstrating that the promiscuous drugs in our data set bind to diverse proteins.

Thus, binding site similarity and structural similarity are the only of the five studied properties that correlate well with the degree of promiscuity. It must be noted that 15% of the target pairs with similar binding sites are dissimilar in global structure.

### Similar Binding Sites for Targets of Methotrexate, Acarbose and Quercetin

**Figure 7:**
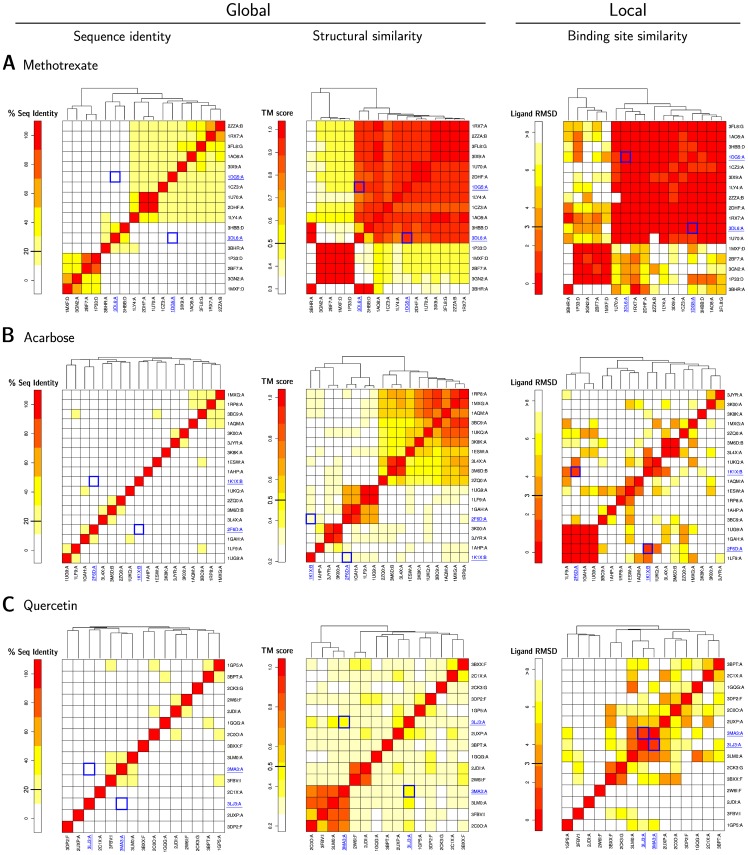
Target similarity. The heatmaps for the targets of methotrexate (A), acarbose (B) and quercetin (C) show that the target sequences are dissimilar (left), the global structural similarity (middle) is comparable to the sequence identity and the binding sites are overall more similar (right). (**A**) Two DHFR (1dg5, 3dl6) with a conserved 3D structure and similar binding sites for methotrexate are highlighted. (**B**) Although the proteins 4-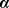-glucanotransferase (1k1x) and glucoamylase (2f6d) have globally distinct sequences and structures, they bind acarbose in a very similar way. (**C**) The two protein kinases PI3KCG (3lj3) and PIM1 (3ma3) share a similar binding pocket for quercetin.

We selected three examples from the highly promiscuous drugs in Table 2 for detailed discussion. The aim was to have three representative approved drugs with targets of low, very low and no pair-wise sequence identity, resulting in methotrexate, acarbose and quercetin. Methotrexate is an antifolate drug, used in the treatment of cancer and autoimmune diseases. It is mainly inhibiting dihydrofolate reductases (DHFR), which are necessary for DNA and RNA synthesis. Acarbose, an alpha-glucosidase inhibitor, is an anti-diabetic drug against type 2 diabetes. Due to its ability of delaying the absorption of carbohydrates from the small intestine, it is also used to treat cardiometabolic disorders. Quercetin is a natural flavonoid with beneficial effects on blood pressure and lipid metabolism. Its inhibitory activity against a wide range of kinases suggested a potential application as anticancer drug. Figure 7 shows three different heatmaps for each of the three drugs. The heatmaps compare the targets in terms of sequence identity, the overall structural similarity (TM-score) and the binding site similarity (LigandRMSD).

**Figure 8:**
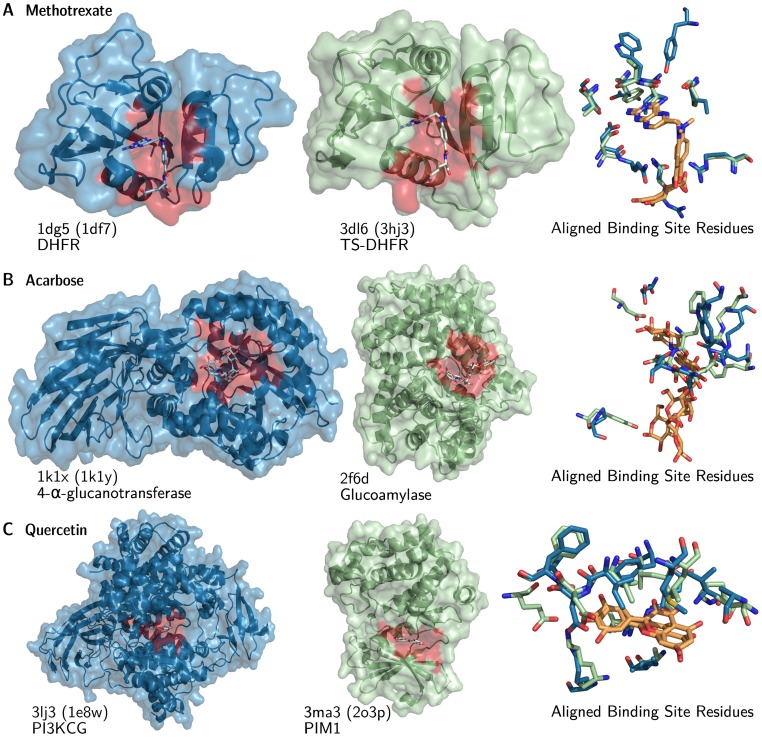
Structural details of binding site similar targets. The binding site alignments for the targets of (**A**) methotrexate, (**B**) acarbose and (**C**) quercetin (highlighted in blue in Figure 7) are visualized. Binding sites are highlighted in red and ligands are displayed in orange. PDB IDs are given below the structures. If the given ID is a representative of a cluster, the PDB ID of the underlying structures is given in parentheses.

For all the three cases, the similarity among the targets is best reflected in the binding site similarity heatmaps. Sequences are not indicative of any relationship between those proteins although they are all binding the same drug. The structural similarity could underline clusters of similar targets but misses interconnections among the groups (for example in Figure 7.A and Figure 7.B), or identifies only small clusters unrelated to each other as in Figure 7.C. Two illustrative target proteins are underlined for each of the drugs with the corresponding structures shown in Figure 8.

The first example – methotrexate – is the approved drug with most similar binding sites (79; see Table 4). Figure 7.A provides an overview of the similarity of its targets and their binding sites. The left heatmap shows that its sequences are clustered in three groups. These correspond to the three groups of targets of the drug (from bottom left to the top right) : Pteridine reductases, thymidylate synthases, and dihydrofolate reductases (DHFRs). The heatmap for global structural similarity in the middle shows a similar picture as the one for sequence identity on the left. However, weak global structural similarities are apparent for all its targets. One single exception is a thymidylate synthase (TS) from E. *coli* (PDB ID 3 bhr), in the bottom left corner, similar to two TS-DHFR (3dl6, 3 hbb). The heatmap for binding site similarity on the right shows two predominant groups : Pteridine reductases (bottom left) and DHFRs plus TSs. However, the separation between those groups is less pronounced, suggesting that the binding site of methotrexate is more conserved than the global structure of the proteins. In human, the binding to DHFR is exploited in cancer therapies, since it is blocking the synthesis of DNA. Methotrexate is targeting DHFRs of other organisms too, such as *Mycobacterium tuberculosis* or *Leishmania major*, where the promiscuous binding to pteridine reductase 1 causes resistance to the drug [51]. Two DHFRs (1dg5, 3dl6) are underlined in blue in Figure 7.A. The structural detail for methotrexate in Figure 8.A shows two dihydrofolate reductases (1dg5, 3dl6) from human pathogens with only 9% sequence identity, but sharing a common 3D structure (TM-score 0.89) and binding site (LigandRMSD 0.46 Å and SMAP P-Value 0). The binding site detail on the right in Figure 8.A shows a very good agreement of the residues.

The 18 targets of acarbose are dissimilar in sequence and mainly clustered in three groups (Figure 7.B) when considering global structural similarity (middle heatmap). The latter picture is resembled by the binding site similarity heatmap on the right, although the pair-wise similarities are fewer. Two proteins, a 4-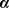-glucanotransferase (1k1x) and a glucoamylase (2f6d) – even though completely different in sequence (Sequence identity 9.7%) and in structure (TM-score 0.32) – are showing a conserved binding site able to bind acarbose (LigandRMSD 1.69 Å, but SMAP P-Value 0.97). The corresponding aligned structures are shown in Figure 8.B with some differences in the amino acid positions of the binding sites, but still having similar physicochemical properties.

The final example (Figure 7.C) shows how inhomogeneous the targets of quercetin are. They are dissimilar in sequence, have a low pair-wise structural similarity (except for two small clusters on the bottom left) but show many pairs with similar binding sites. In particular, two protein kinases PI3KCG (3lj3) and PIM1 (3ma3), are well known targets of quercetin and implicated in cancer cell biology [52]. They show only 8% sequence identity, a TM-score of 0.46 indicating no structural similarity but their binding sites are very similar (LigandRMSD 1.41 Å, but SMAP P-Value 0.28). The binding site residues align structurally and match physicochemically very well, although they mostly mismatch on the sequence level.

### Conclusion

Finding general reasons for drug promiscuity is still an open problem. Nine studies – mostly from pharmaceutical companies – draw partly inconsistent conclusions on the influence of a drug’s molecular weight and its hydrophobicity on drug promiscuity. We contributed to this discussion by pursuing for the first time a comprehensive structural approach and by investigating two more possible sources for drug promiscuity: ligand flexibility and binding site similarity. However, our drug data set is limited in size due to the restriction to PDB.

We analyzed a structural dataset of 164 promiscuous drugs bound to 712 unique protein targets from the PDB. We found no correlation to weight or hydrophobicity and a weak correlation to ligand flexibility. However, we found some correlation (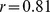, 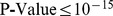 ) between the number of targets and the number of similar binding sites. Thus, our analysis supports [22], [23], [26] that molecular weight is not indicative of promiscuity and it contradicts the findings presented in [19]–[21]. Regarding hydrophobicity, the studies mostly agree that hydrophobic compounds tend to be more promiscuous. However, Shoichet and co-workers argue that many drug-target associations from high throughput screenings are biased by hydrophobic drug aggregation [28], [29]. Our finding that there is no correlation between the degree of promiscuity and hydrophobicity supports this view but does not reflect the findings in [19], [20], [22]–[26]. However, it must be noted that our dataset is limited to the PDB and thus has an intrinsic bias. This limitation directly affects the investigated number of drugs. Thus, the comparability to the other studies of drug promiscuity is limited in that sense. A big portion of drugs binds for example to membrane proteins like GPCRs [34], which are underrepresented in the PDB. Thus, the trends in terms of physicochemical properties reported in the discussed studies might be driven by the nature of drugs binding to membrane proteins.

None of the discussed studies considered a structural analysis of the ligands and/or their protein binding sites. To this end, we clustered the drug conformers and compared all binding sites. As a result, we found a weak correlation of the degree of drug promiscuity to ligand flexibility (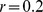), a correlation to structural similarity (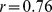) and even higher to the number of similar binding sites (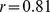). Furthermore, we found that for 71% of the drugs at least one pair of their targets’ binding sites is similar and for 22% all are similar.

Thus, we conclude that binding site similarity is the most important prerequisite for a promiscuous PDB drug to bind to multiple PDB targets and that ligand flexibility has a minor impact. Molecular weight and hydrophobicity do not seem to influence whether a drug is promiscuous or not. It is important to note that structural similarity gives a strong correlation as well. However, global structural similarity is also reflected in the pairs of similar binding sites but misses the important examples of similar binding sites in globally structural dissimilar proteins. In particular, 15% of all target pairs with a similar binding site are dissimilar in global structure and would have not been detected by other approaches on sequence or global structure level.

Our study demonstrates that it is vital and worthwhile to incorporate structural data in drug discovery pipelines and that the efforts in structural genomics and algorithm development for structural bioinformatics have to be strengthened. As supported by our findings, protein local structural alignments bare a huge potential to infer so-far unknown drug-target relationships. Apart from identical ligands, as in this study, LigandRMSD is suitable to score binding site alignments with dissimilar ligands as well. This is achieved by a maximum common substructure detection in a pair of ligands. The further mapping of the drug-protein interaction space with structural bioinformatics approaches – such as the approach used in this study – gives hypotheses for drug repositioning or off-target detection, speeding up drug development and uncovering causes for adverse drug reactions. Analyses from a convergent evolution point of view, such as the detection of protein interaction interface mimicry strategies by viruses, can also be driven by local structural alignments.

## Methods

### Drug-Target Dataset

#### Drugs

We integrated 3042 drugs from TTD [53] (version 4.3.0.1, 2011/07/01), 1261 from the Comparative Toxicogenomics Database (CTD) [54] (2011/07/05), and 1348 approved drugs from DrugBank [55] (version 3, 2011/11/28) leading to a set of 3551 drugs. All drugs were mapped to PubChem [56] CIDs, using a provided mapping or the PubChem Power User Gateway (PUG). In the latter case, the structures were first transformed to InChI and SMILES using OpenBabel (version 2.2.3, openbabel.sourceforge.net) [57] and Pybel [58]. Only for PDB ligands, the provided SMILES were used. These string representations of the chemical structures were then sent to the PubChem PUG web service, which returns the corresponding PubChem CID. If no CID was returned (because the query structure is not present in PubChem), a similarity search via the PUG is performed. This was implemented as a binary search for the highest possible Tanimoto similarity to the query structure. Only compounds with a Tanimoto score ≥0.9 were considered for the analyses in this work. If the search result comprises more than one compound, the smallest CID is retained as a representative.

#### Promiscuous drugs

We obtained 10430 co-crystallized ligands from 66820 structures in the PDB [31] (accessed 2010/10/29). Of these 10430, 560 are drugs present in the set above, 8977 are non-drugs and 893 were blacklisted. Ligands were blacklisted if they are small compounds (five non-hydrogen atoms or less), common cofactors, detergents or solutes or the compounds listed in [59]. The filtering of small compounds was performed with OpenBabel. For each PDB structure, all of its ligands were extracted using mmLib [60] with Python.

#### Protein targets

Subsequently, all PDB protein structures were clustered according to the 95% sequence identity clusters as provided by the PDB, resulting in a set of non-redundant targets (i.e. the cluster representatives). The number of targets as listed throughout this paper is based on this non-redundant set of protein structures for a drug. Out of the 560 drugs with at least one target in PDB, 164 have three or more (non-redundant) targets. These 164 are the set of promiscuous drugs used throughout the paper. The 164 drugs are present as ligands in a total of 2284 PDB structures, clustered to 712 (non-redundant) protein targets.

### Binding Site Alignment

The binding sites of all promiscuous drugs (three or more different targets) were aligned with the binding site alignment tool SMAP (version 2.0, funsite.sdsc.edu, see Table 5 for the setup ) [15], [45], [46].

SMAP uses a Cα representation of the protein structure, characterizing each such atom by a geometric potential to reflect the distance to the surface and neighboring atoms [45]. Two protein binding sites are aligned by computing the maximum weight common subgraph of the graphs built from a tessellation of Cα atoms (nodes). Weights are amino acid frequency profile distances. The alignment score for the aligned residue pairs is computed from their profile distances, weighted by distance and normal vector differences [46]. A P-Value is computed from an estimated background probability distribution of binding site alignment scores for a pair of aligned ligand binding sites [61]. A typical P-Value threshold is 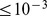 as in two studies of the algorithm authors [37], [62].

An alignment of two binding sites was considered to be significant only if their bound ligands were in a similar position (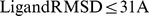).

Only ligand binding sites as found in PDB structures were considered during the alignment by setting the appropriate option in the SMAP configuration file (see Table S1 for the detailed setup). For each drug 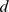 (targeting proteins 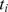) and for each pair of non-redundant targets (i.e. cluster representatives) 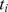 and 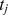, we compared the (redundant) structures 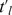 against all (redundant) structures for 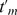 (i.e. 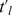 and 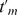 are cluster members represented by 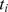 and 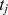, respectively). If at least one of these pairs 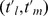 has a 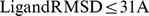, then the binding sites in 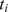 and 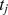 are considered similar.

For the drug 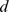 bound by each member of a pair of aligned binding sites (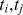), the LigandRMSD of the two drug conformers 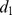 and 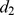 was calculated as follows: First, the maximum common subgraph 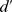 of 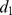 and 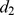 was computed using OpenBabel, or the Small Molecule Subgraph Detector [49] if no isomorphism was found by OpenBabel. The RMSD between the conformers 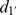 and 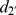 in 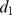 and 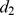 was computed for the positions according to the SMAP binding site alignment (RMSD′) and for the optimal alignment of the two ligands (RMSD”). For details on the computation of the RMSD” see section “Comparison of Ligand Conformers”). Finally, the LigandRMSD is given by 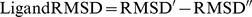.

Binding site alignments for the 164 promiscuous drugs with the 712 non-redundant targets were computed and scored with LigandRMSD. Thus, the comparison of all targets for each of the 164 drugs resulted in 38244 local structure alignments.

### Sequence and Structure Alignment, Protein Families

For each structural binding site alignment, the corresponding protein sequence alignment was computed as a global alignment, using an implementation of the Needleman-Wunsch algorithm (program needle) in the EMBOSS suite [63] (version 6.1.0). Gap penalties were set to 10 for gap opening and 0.5 for gap elongation. BLOSUM62 was used as substitution matrix. Sequences were extracted from the FASTA file provided by the PDB as of 2010/10/29.

Global structural alignments were performed using TM-align [50] with default parameters on single PDB chains.

Protein families were assigned to the target proteins using Pfam release 26.0 [35].

### Comparison of Ligand Conformers

To explore the conformational space of the drugs in the PDB, all drugs were identified and extracted from the 2284 PDB files. A python script together with Pybel [58] was used to perform this task. Subsequently, for each drug 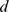 and each pair of its conformers 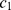 and 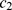, the conformers were superimposed (3D rigid structure alignment) onto each other using OpenBabel and RDKit for Python (rdkit.org). The lowest RMSD from both of the methods was retained. The RMSD of the superposition of two conformers 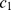 and 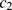 is their distance during the clustering. Next, the conformers were clustered with hierarchical clustering using average linkage. The hierarchical tree of clusters was turned into distinct clusters by cutting off at 1.4 Å. The threshold of 1.4 Å RMSD allows to find well matching conformers by staying well below 2.5 Å as used in docking studies [64].

### Drug Physicochemical Properties

Drug physicochemical properties were computed using the QSAR descriptors of MOE (Molecular Operating Environment, version 2010.10, Chemical Computing Group Inc.). The computed octanol-water partition coefficient (log *P*, a measure for the hydrophobicity of a compound) and the number of rotatable bonds was computed. The relative number of rotatable bonds represents the ratio of rotatable bonds to the total bond count. Bonds in a ring are not counted as rotatable.

### Computation, Visualization and Availability

All computations performed for this work – if not stated otherwise – were scripted in Python using among others Pybel [58], BioPython (biopython.org), mmLib [60] and RDKit packages on Linux 2.6.

Protein structures were visualized with PyMol (pymol.org).

The R environment for statistical computing and graphics software package (R-project.org) was used for data evaluation and to create the plots shown in this work. To generate the heat maps, the Heatplus package from the Bioconductor tools for R (bioconductor.org) was used.

Pearson correlation coefficients 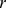 were computed and tested for statistical significance using the R function cor.test. A low P-Value≤0.05 denotes a low probability of the true correlation being equal to 0.

A linear regression model was computed for the combination of all studied drug properties (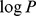, molecular weight, conformer count, relative rotatable bond count and rotatable bond count) using the R function lm.

The distributions are shown as density plots, being a smoothed approximation generated with the R function density. To assess the difference in a pair of distributions of original data, the Kolmogorov-Smirnov test was used. A high P-Value>0.05 is indicative of similar distributions.

The result data for this study are available at: http://www.biotec.tu-dresden.de/research/schroeder/publications/2012_Drug_Promiscuity_PDB_Suppl/. A description of the files is given in the Supporting Information S1.

## Supporting Information

Figure S1 Density plot of the sequence identity distribution for all pairs of proteins binding the same drug. The distribution for protein pairs with similar binding sites is shown in green. Half of the similar binding site pairs have a sequence identity ≤23% and 25% have a sequence identity ≤15%%. The sequence identity density maximum for all pairs is at 9% (mean 19%) and for the similar binding site pairs at 22% (mean 28%).
(TIFF)

Figure S2 Density plots of the molecular weight distribution for the promiscuous drugs (green) and for all drugs in the PDB. The underlying distributions are similar (Kolmogorov-Smirnov test P-Value = 0.581).
(TIFF)

Figure S3 Density plots of the computed log distribution for the promiscuous drugs (green) and for all drugs in the PDB. The underlying distributions are similar (Kolmogorov-Smirnov test P-Value = 0.1255).
(TIFF)

Figure S4 **The absolute number of rotatable bonds for different promiscuous drugs.** Green solid dots denote the mean.
(TIFF)

Figure S5 Density plots of the rotatable bond count distribution (relative to the total number of bonds) for the promiscuous drugs (green) and for all drugs in the PDB. The underlying distributions are dissimilar (Kolmogorov-Smirnov test P-Value = 0.007).
(TIFF)

Figure S6 The number of rotatable bonds (relative to the total number of bonds) for different promiscuous drugs. Overall, the relative rotatable bond count drops with increasing number of targets. Green solid dots denote the mean.
(TIFF)

Figure S7 Histogram showing the RMSDs of all conformers of a drug against each other.
(TIFF)

Figure S8 Histogram of the LigandRMSDs for the binding site alignments of the promiscuous drug targets. A LigandRMSD of ≤3 Å represents similar ligand conformers.
(TIFF)

Figure S9 Density plot of the TM-score distribution for all pairs of proteins binding the same drug. The distribution for protein pairs with similar binding sites is shown in green. 15% of the similar binding site pairs are significantly dissimilar in global structure with a TM-score <0.5. The median TM-score is 0.43.
(TIFF)

Table S1 The parameters of SMAP.
(PDF)

Supporting Information S1 Description of supplementary files. The files list the detailed drug physicochemical properties and the results of the protein (local) alignments.
(PDF)
