# Correction: Drug Promiscuity in PDB: Protein Binding Site Similarity Is Key

**DOI:** 10.1371/annotation/0852cc69-8cea-4966-bb8a-ae0b348d1bd9

**Published:** 2013-12-20

**Authors:** V. Joachim Haupt, Simone Daminelli, Michael Schroeder

In the Method section, there are two errors in the subsection titled “Binding Site Alignment.” "LigandRMSD ≤ 31 A" appears twice incorrectly. To see the correct version, please view here: http://plosone.org/corrections/pone.0065894.s001.cn.tif

In the 6th paragraph under the heading “Promiscuous Drugs: Hydrophobicity vs, Molecular Weight, ” there is an error in the reference. The correct reference should read: Hann et al.

There is an error in the caption of Figure S1, which includes an additional percent sign at the end of the third sentence. The third sentence should read: Half of the similar binding site pairs have a sequence identity ≤23% and 25% have a sequence identity ≤15%.

