# Supplementary figures and images for: Drug Promiscuity in PDB: Protein Binding Site Similarity Is Key
Source: PLoS One. 2013 Jun 21;8(6):e65894. doi: 10.1371/journal.pone.0065894 (PMC3689763; doi:10.1371/journal.pone.0065894)

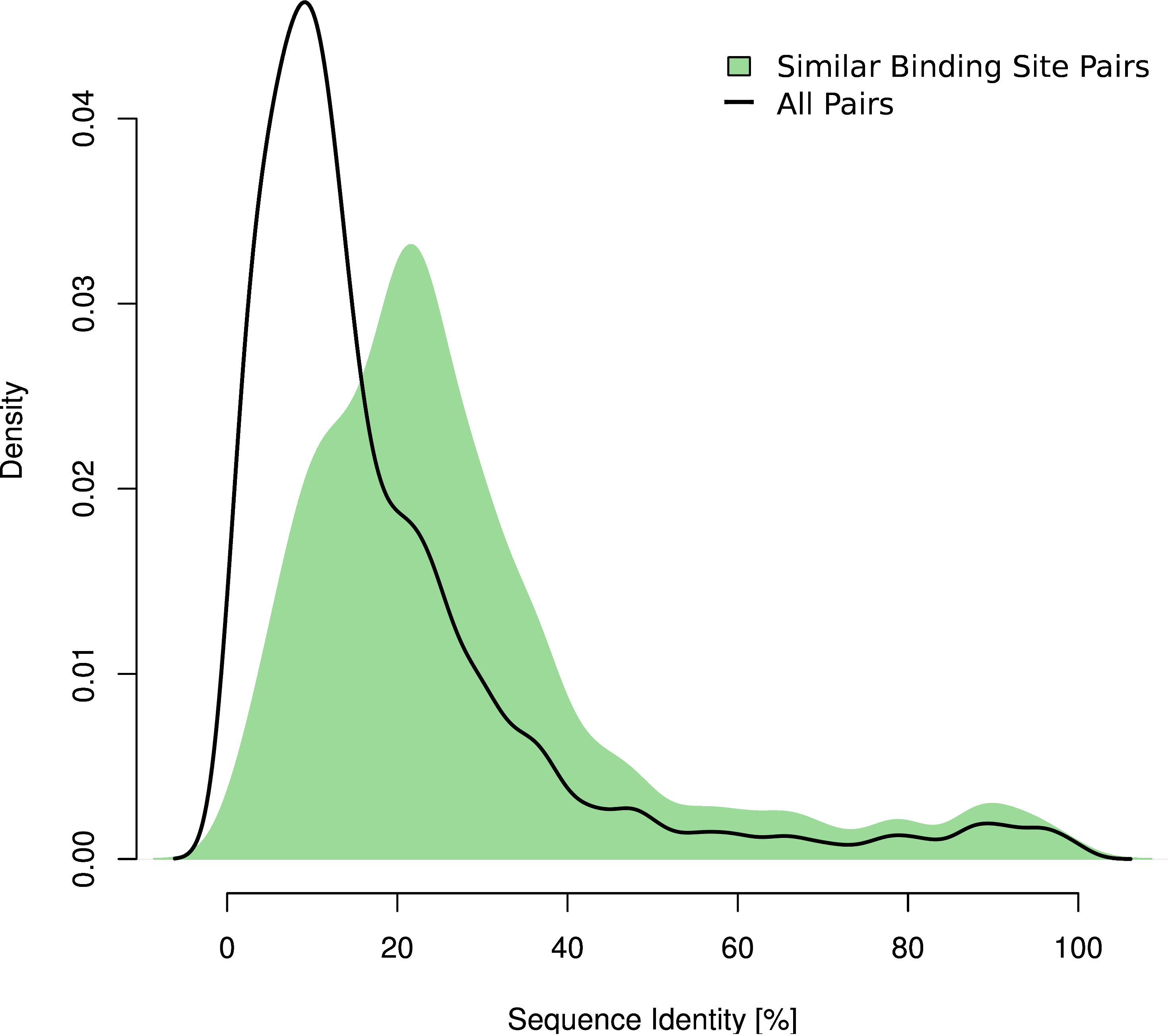

Supplement: Figure S1 — Density plot of the sequence identity distribution for all pairs of proteins binding the same drug. The distribution for protein pairs with similar binding sites is shown in green. Half of the similar binding site pairs have a sequence identity ≤23% and 25% have a sequence identity ≤15%%. The sequence identity density maximum for all pairs is at 9% (mean 19%) and for the similar binding site pairs at 22% (mean 28%). (TIFF) [file pone.0065894.s001.tiff]

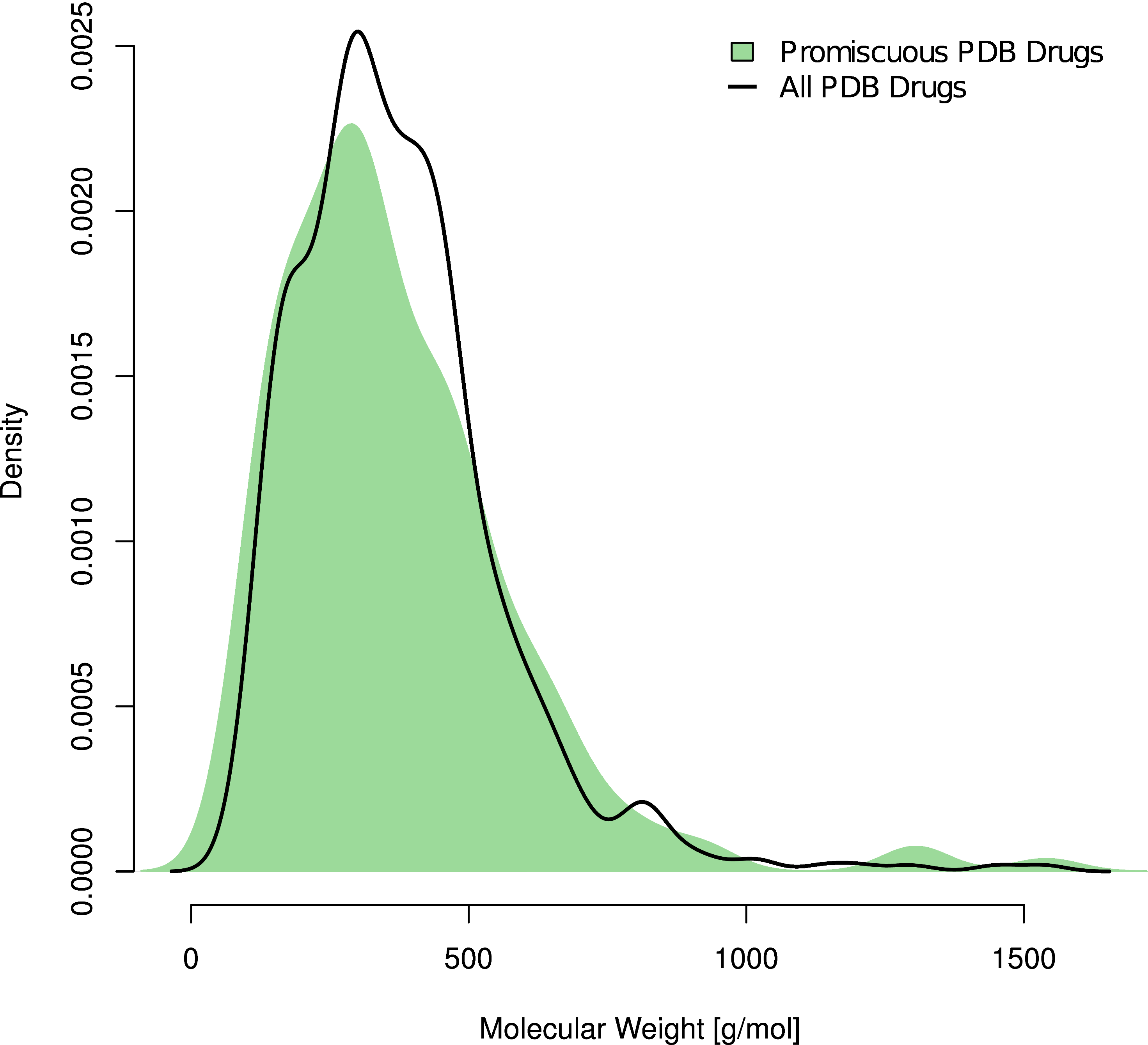

Supplement: Figure S2 — Density plots of the molecular weight distribution for the promiscuous drugs (green) and for all drugs in the PDB. The underlying distributions are similar (Kolmogorov-Smirnov test P-Value = 0.581). (TIFF) [file pone.0065894.s002.tiff]

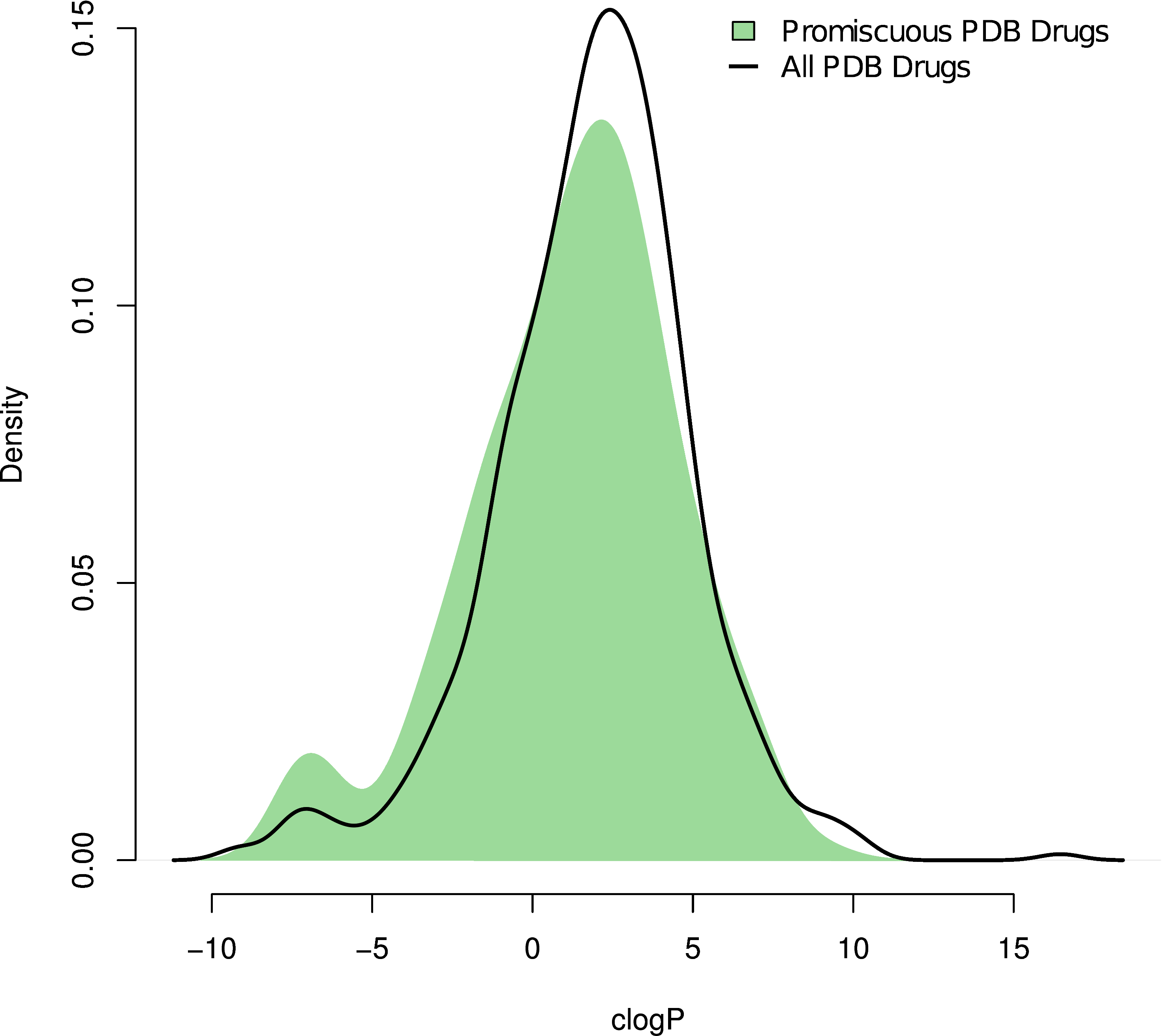

Supplement: Figure S3 — Density plots of the computed log distribution for the promiscuous drugs (green) and for all drugs in the PDB. The underlying distributions are similar (Kolmogorov-Smirnov test P-Value = 0.1255). (TIFF) [file pone.0065894.s003.tiff]

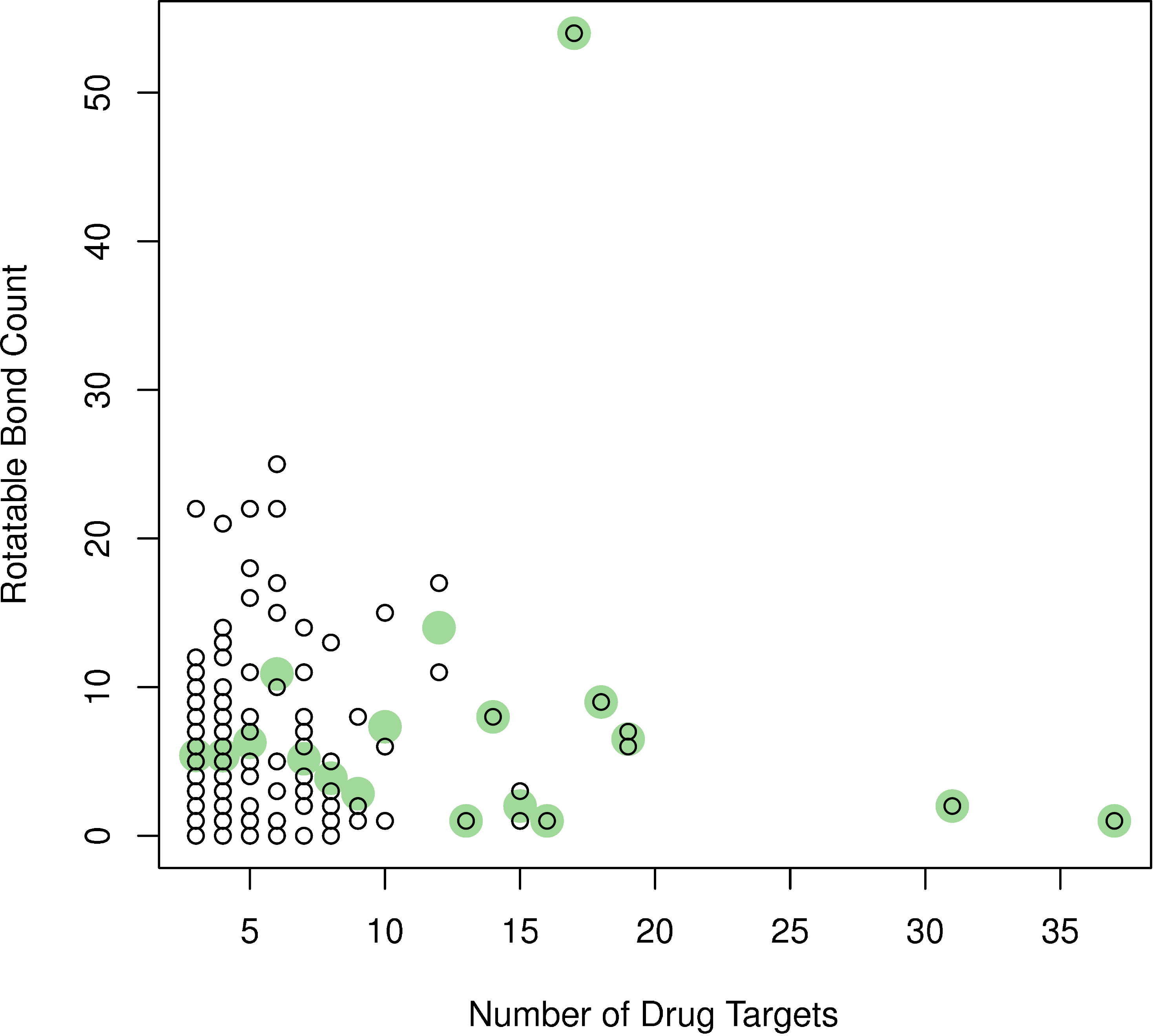

Supplement: Figure S4 — The absolute number of rotatable bonds for different promiscuous drugs. Green solid dots denote the mean. (TIFF) [file pone.0065894.s004.tiff]

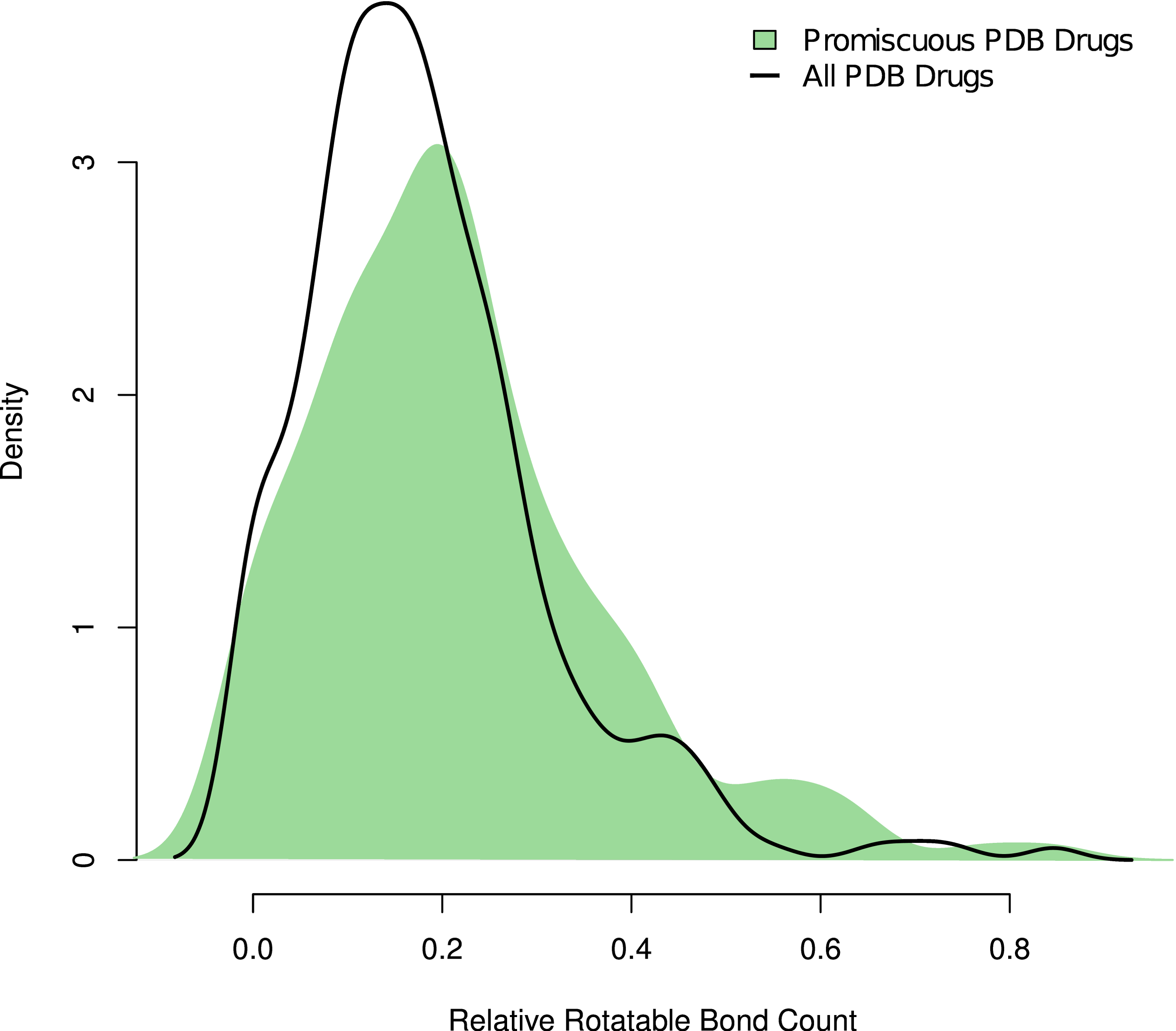

Supplement: Figure S5 — Density plots of the rotatable bond count distribution (relative to the total number of bonds) for the promiscuous drugs (green) and for all drugs in the PDB. The underlying distributions are dissimilar (Kolmogorov-Smirnov test P-Value = 0.007). (TIFF) [file pone.0065894.s005.tiff]

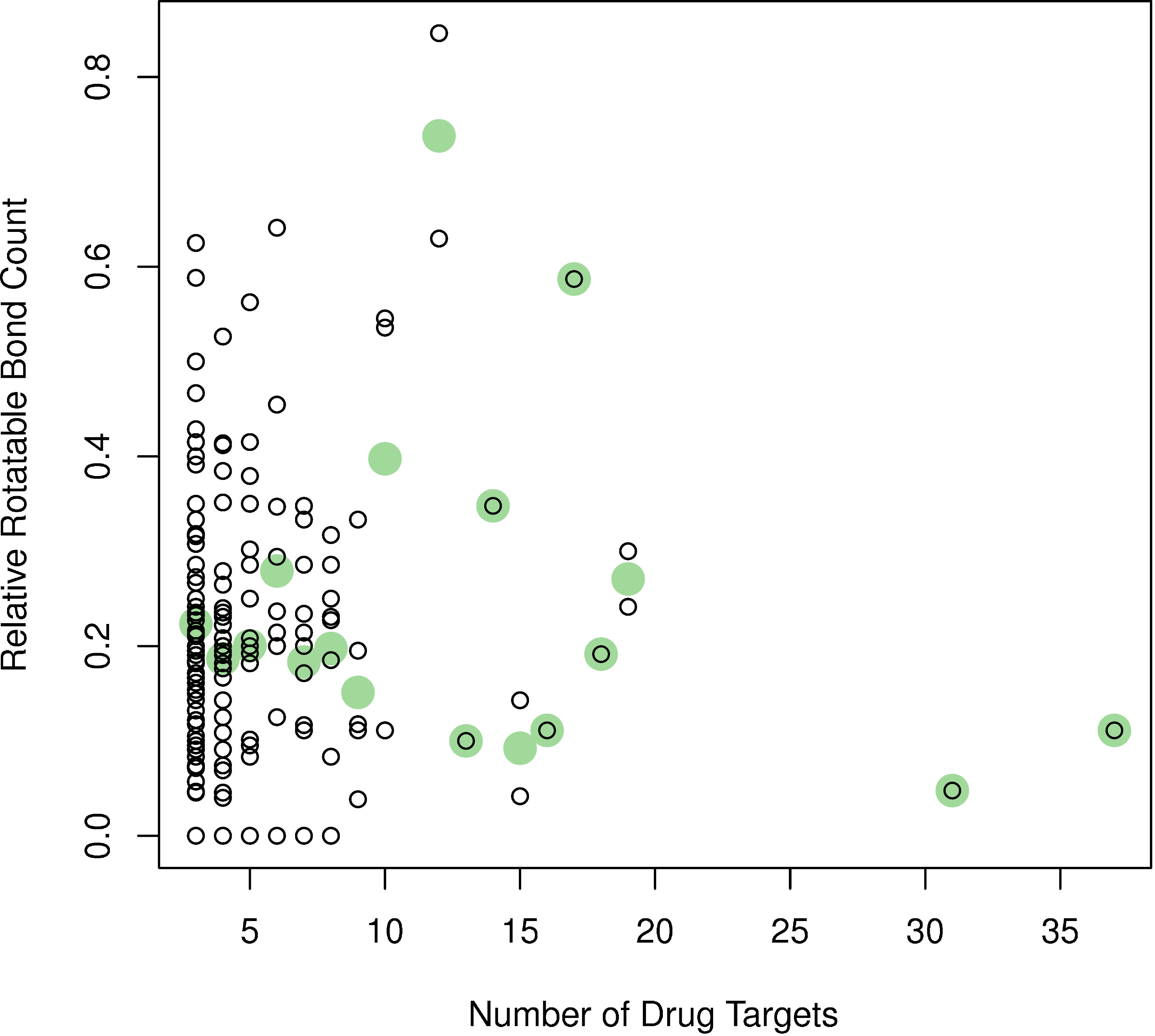

Supplement: Figure S6 — The number of rotatable bonds (relative to the total number of bonds) for different promiscuous drugs. Overall, the relative rotatable bond count drops with increasing number of targets. Green solid dots denote the mean. (TIFF) [file pone.0065894.s006.tiff]

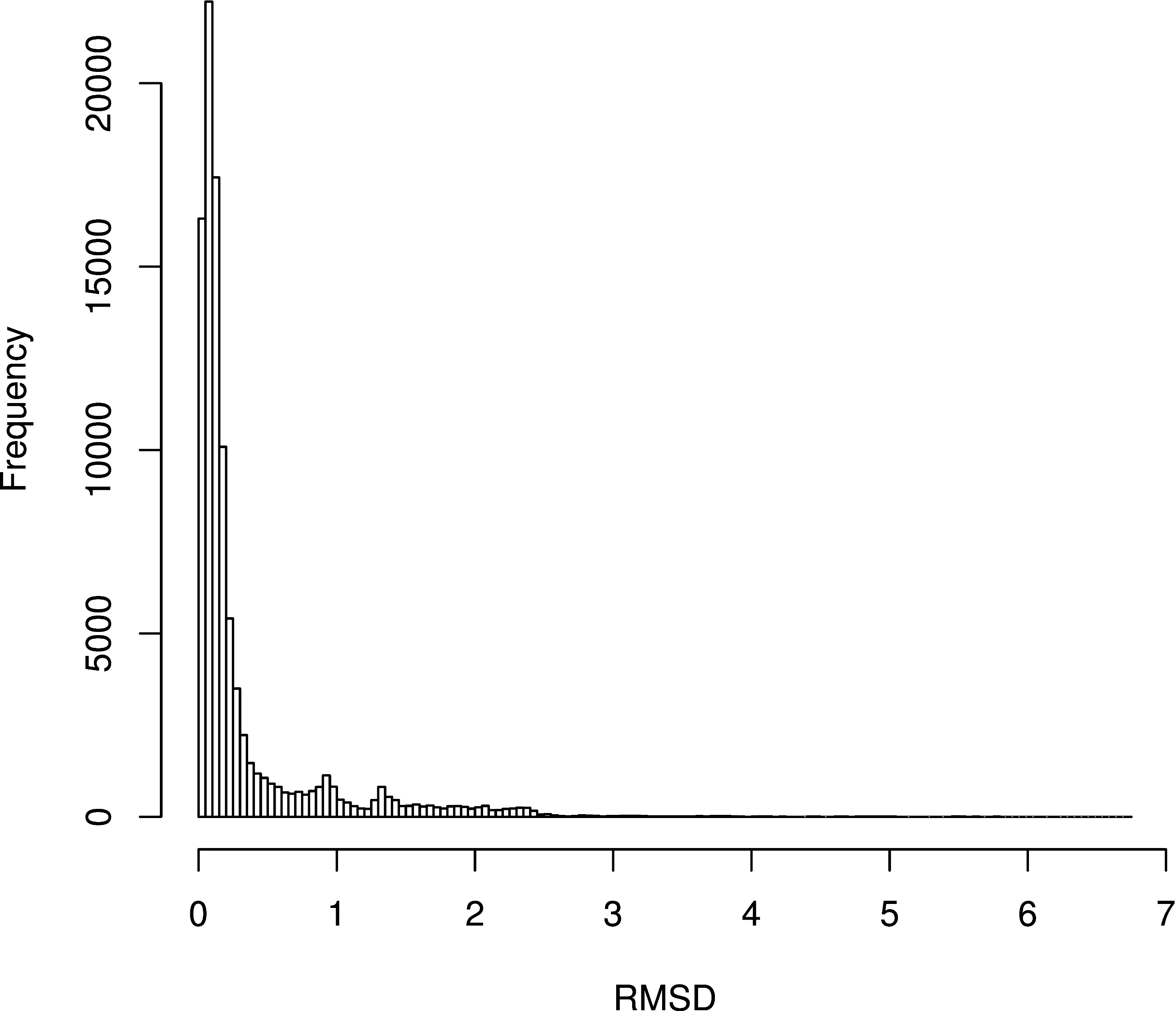

Supplement: Figure S7 — Histogram showing the RMSDs of all conformers of a drug against each other. (TIFF) [file pone.0065894.s007.tiff]

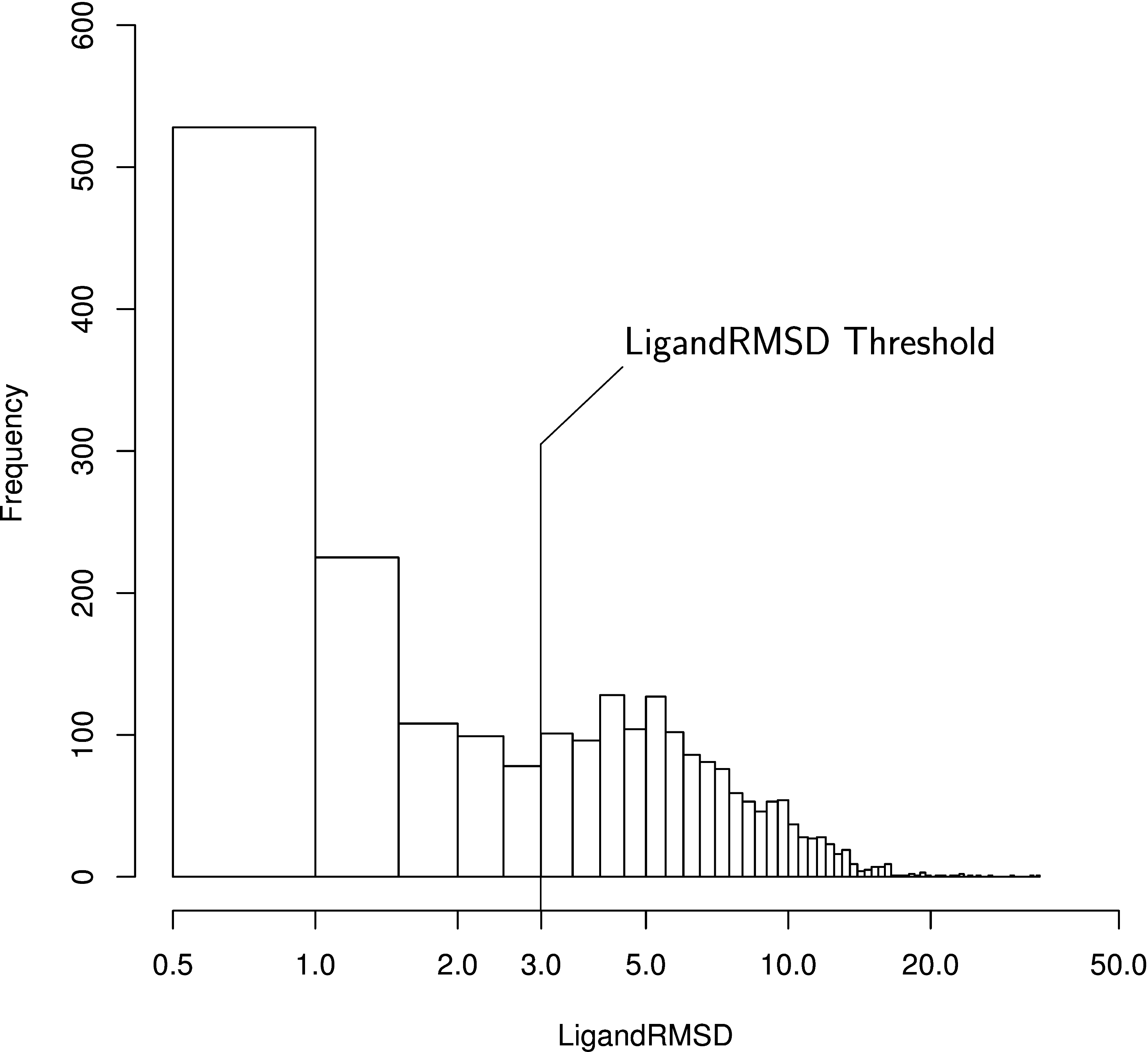

Supplement: Figure S8 — Histogram of the LigandRMSDs for the binding site alignments of the promiscuous drug targets. A LigandRMSD of ≤3 Å represents similar ligand conformers. (TIFF) [file pone.0065894.s008.tiff]

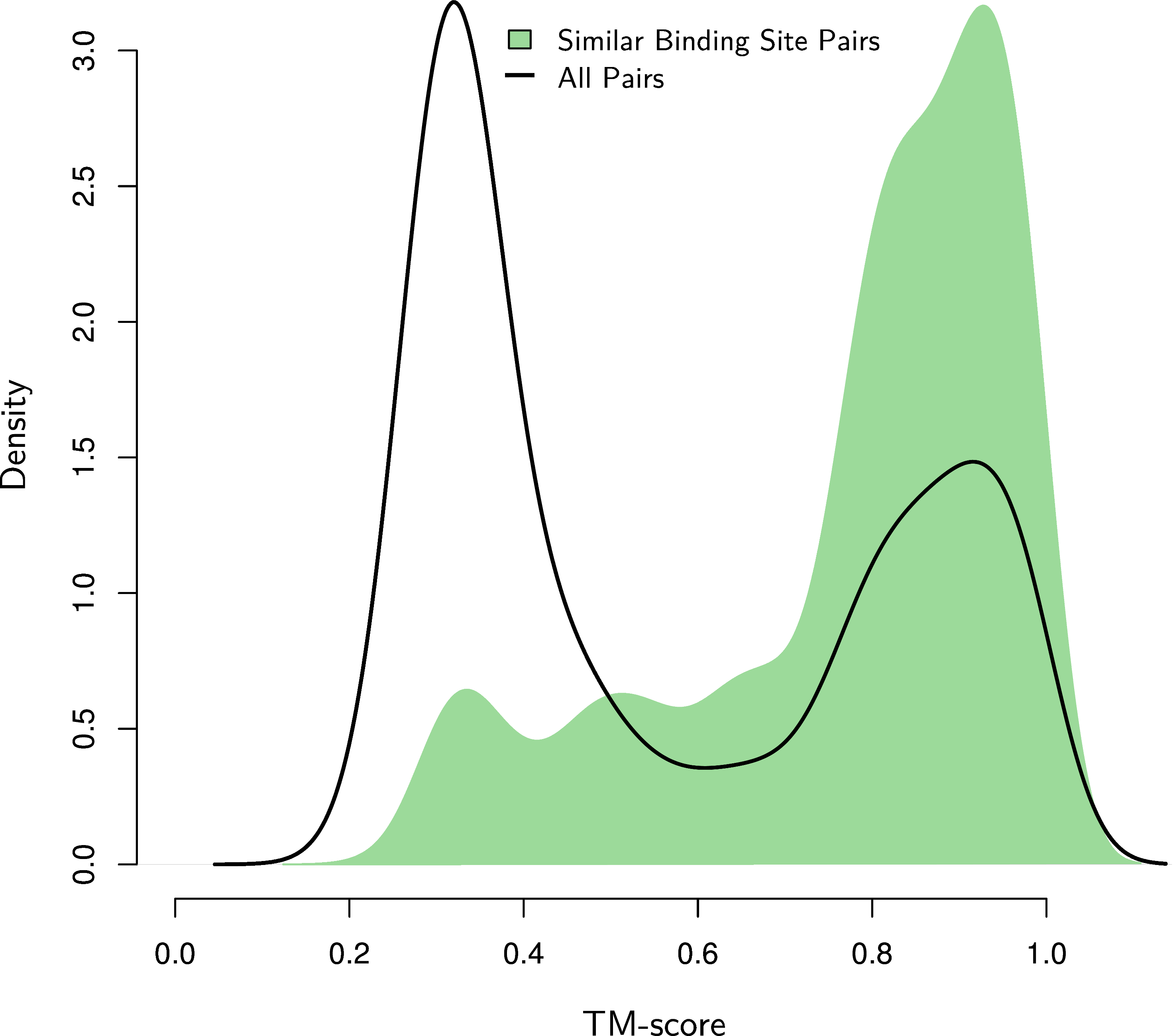

Supplement: Figure S9 — Density plot of the TM-score distribution for all pairs of proteins binding the same drug. The distribution for protein pairs with similar binding sites is shown in green. 15% of the similar binding site pairs are significantly dissimilar in global structure with a TM-score <0.5. The median TM-score is 0.43. (TIFF) [file pone.0065894.s009.tiff]
