# Supplementary material for: Drug Promiscuity in PDB: Protein Binding Site Similarity Is Key
Source: PLoS One. 2013 Jun 21;8(6):e65894. doi: 10.1371/journal.pone.0065894 (PMC3689763; doi:10.1371/journal.pone.0065894)
Supplement: Table S1 — The parameters of SMAP. (PDF) [file pone.0065894.s010.pdf]

Table S1. The parameters of SMAP.

Parameter	Value	
ASSOCIATE_GRAPH_NODE_FILTER	0.4	*
LIGAND_CONTACT_DISTANCE_CUTOFF	5.0	*
LOCAL_SCORE	true	
MATCH_SECONDARY_STRUCTURE	false	*
MAX_ATOM_SPHERE_RADIUS	5.0	
MAX_CA_SPHERE_RADIUS	7.5	
MAX_NUM_PL	5.0	
MIN_ATOM_SPHERE_DISTANCE	3.0	
MIN_CA_SPHERE_DISTANCE	5.0	
MIN_PL_ATOM_SPHERE_SIZE	20.0	
MIN_PL_CA_SPHERE_SIZE	5.0	
PVALUE_CUTOFF	1.0	*
QUERY_LIGAND_ID	<current ligand>	*
QUERY_LIGAND_SITE_ONLY	true	*
SCORE_MATRIX	McLACHLAN	
TEMPLATE_LIGAND_ID	<current ligand>	*
TEMPLATE_LIGAND_SITE_ONLY	true	*
TIMES_RANDOM_SHUFFLE	0.0	

Non-default parameters are indicated with an asterisk (*).
