# Supplementary material for: Drug Promiscuity in PDB: Protein Binding Site Similarity Is Key
Source: PLoS One. 2013 Jun 21;8(6):e65894. doi: 10.1371/journal.pone.0065894 (PMC3689763; doi:10.1371/journal.pone.0065894)
Supplement: Supporting Information S1 — Description of supplementary files. The files list the detailed drug physicochemical properties and the results of the protein (local) alignments. (PDF) [file pone.0065894.s011.pdf]

Supporting Information S1

File	Description
drug_properties.csv	This file lists all promiscuous drugs and physicochemical properties like number of rotatable bonds, octanol-water partition coefficient, molecular weight, the number of conformer clusters and the total number of observed conformers in all PDB structures. The PDB Chemical ID and the corresponding PubChem CID is given for each drug. A CID might represent more than one PDB Chemical ID. See Methods for details.
drug_target_pairs_bs_sim.csv	This file lists all drug-target pairs of promiscuous drugs used for the binding site alignment. For each PDB chain, the cluster representative and the CATH classifications are given. The P-Value of the SMAP binding site comparison, the LigandRMSD, and the sequence identity is given for each pair.
aligned_res_successful.csv	This file lists all aligned residues for each successful alignment in drug_target_pairs_bs_sim.csv.

The files are available at: http://www.biotec.tu-dresden.de/research/schroeder/publications/2012_Drug_Promiscuity_PDB_Suppl/.
